# The n-type Ge photodetectors with gold nanoparticles deposited to enhance the responsivity

**DOI:** 10.1186/1556-276X-9-640

**Published:** 2014-11-27

**Authors:** Hao-Tse Hsiao, I-Chih Ni, Shien-Der Tzeng, Wei-Fan Lin, Chu-Hsuan Lin

**Affiliations:** 1 Department of Opto-electronic Engineering, National Dong Hwa University, Shoufeng, Hualien 974, Taiwan; 2 Department of Physics, National Dong Hwa University, Shoufeng, Hualien 974, Taiwan

**Keywords:** AuNP, Ge photodetector, Responsivity

## Abstract

Gold nanoparticles (AuNPs) have been deposited on n-type Ge photodetectors to improve the responsivity. Two different coverage ratios, including 10.5 and 30.3% of AuNPs have been prepared, and the fabricated photodetectors are compared with the control sample. The 1,310-nm responsivities at -2 V of the control, 10.5% AuNPs, and 30.3% AuNPs samples are 465, 556, and 623 mA/W, respectively. The AuNPs could increase the responsivities due to the plasmon resonance. The reflectance spectra of these samples have been measured to verify that plasmon resonance contributes to the forward scattering of incident light. The reflectance decreases with AuNP deposition, and a denser coverage results in a smaller reflectance. The smaller reflectance indicates more light could penetrate into the Ge active layer, and it results in a larger responsivity.

## Background

Photodetectors are widely used in various fields in our daily life [1,2]. The detection wavelength of a photodetector depends on the choice of the active semiconductor material. Si is a common semiconductor material for use in photodetectors due to its abundant existence on Earth. The bandgap of Si is 1.12 eV, which corresponds to a cutoff photodetection wavelength of 1,100 nm. Visible light and one of the common bands of near infrared, 850 nm, can be detected well by Si. However, other important near-infrared bands, including 1,310 and 1,550 nm, cannot be absorbed by Si [3]. The low-loss and low-dispersion characteristics of infrared light in fiber at these wavelengths make these wavelengths necessary in optical communication [4,5]. Ge, an element in the same group IV as Si, has a smaller bandgap of 0.66 eV, and it can be used for light detection at these wavelengths [6,7]. Various approaches have been tried to boost the photoresponses (responsivities) of Si photodetectors [8-11]. Among them, the investigation of the use of metallic nanoparticles has grown rapidly in the last decade [11-14]. However, few studies have touched on the topic of photoresponse enhancement of Ge based on metallic-nanoparticle incorporation [15]. Metallic nanoparticles on a semiconductor could contribute to the plasmonic interaction, which could result in forward scattering of incident light into the semiconductor to enhance the photoresponse [15,16]. Such plasmon resonance can be used in solar cells, although it may upgrade the (quantum) efficiency at certain wavelengths while degrading at other wavelengths [17]. As compared to applications on solar cells, where broadband utilization is needed, photodetectors could benefit more from plasmonic interaction, since only improvement of a certain wavelength is needed. In this manuscript, gold nanoparticles (AuNPs) have been deposited on the Ge photodetector. The reflectance and responsivities of n-type Ge photodetectors with and without AuNPs covering will be compared to show the enhancement contributed by AuNPs.

## Methods

### Experimental details

#### AuNPs preparation

**Figure 1:**
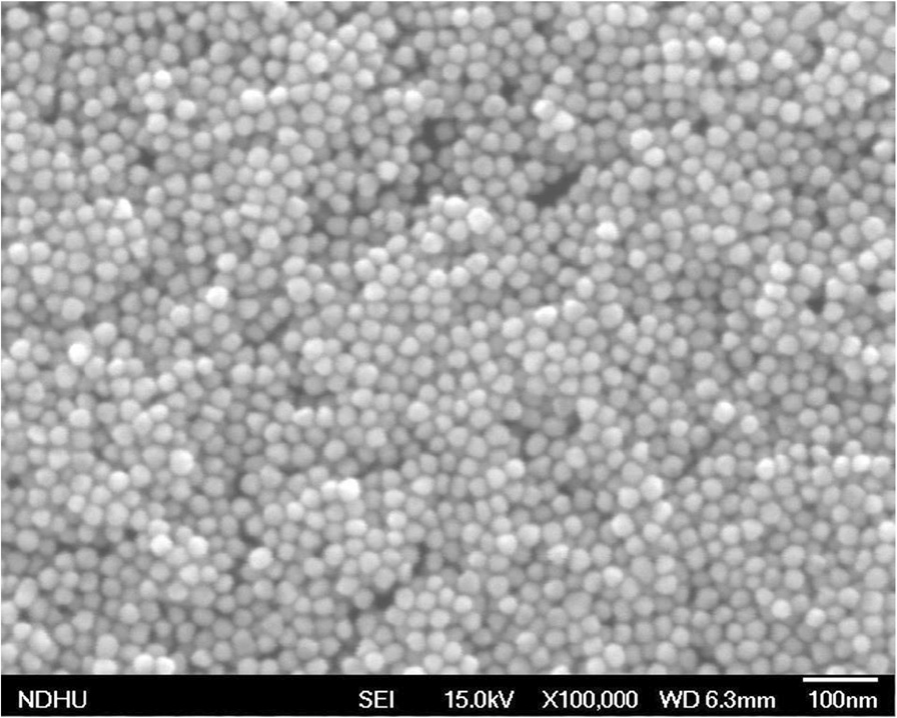
**The field-emission scanning electron microscope photograph of AuNPs.** The size of AuNPs was 30 ± 3 nm, and the shape was close to spherical.

First of all, AuNPs should be prepared in order to deposit them on Ge substrates. The solution of AuNPs was obtained by the method of gold salt reduction [18,19]. Trisodium citrate (TSC) and tannic acid (TA) were used to deoxygenate HAuCl_4_ into AuNPs. With 0.5 mL TA (1%) added to 1-L solution, the 12-nm AuNPs were first formed. If HAuCl_4_ and TSC could be continuously added, the particle size could be enlarged. In this manuscript, the AuNPs with a size of ~30 nm were used. In order to obtain more precise estimation of the sizes and shapes of AuNPs, dense AuNPs were photographed by field-emission scanning electron microscope (FESEM) as shown in Figure 1. The size of AuNPs was 30 ± 3 nm, and the shape was close to spherical. The AuNPs should be modified by 8-mercaptooctanoic acid (MOA) to cause the nanoparticles to become negatively charged in order to avoid agglomeration in the solution. These modification molecules had two terminals. One terminal was the sulfur atom, which could be easily bonded with the AuNP. The other terminal was the functional group of -COOH. NaOH was added to increase the pH value, and the functional groups of -COOH were transformed into -COO^-^. AuNPs attached by negatively-charged -COO^-^ groups were repulsive, and hence, agglomeration could be avoided. The pH value would be a critical parameter influencing the distribution and density of deposited AuNPs on substrates since the amount of negative charge would affect agglomeration and precipitation in the following steps.

**Figure 2:**
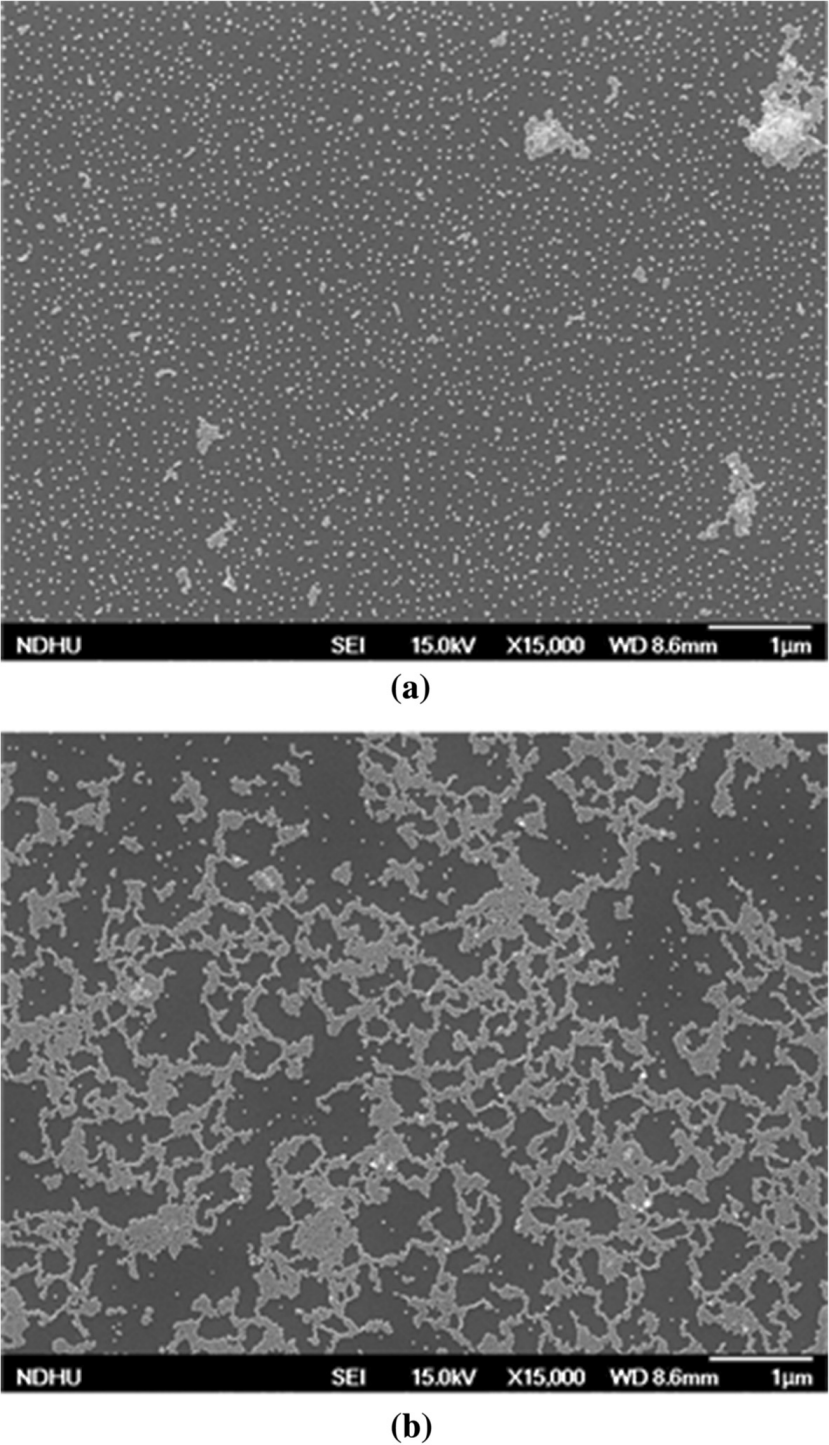
**The AFM images of (a) 10.5% and (b) 30.3% AuNPs on the Ge substrates.** The pH value of the AuNP solution before coating is a critical parameter influencing the coverage density of AuNPs on Ge substrates.

AuNPs could be coated on n-Ge by the method of centrifugation. Before centrifugation, a specific centrifuge tube was prepared. The polydimethylsiloxane (PDMS) was put and tilted at a certain angle in the bottom of the tube in order to provide a vertical surface in the procedure of centrifugation [18]. Subsequently, the Ge substrate was put on the top of PDMS, and the solution of AuNPs was added into the tube. During centrifugation, the AuNPs in solution were forced to bond onto the Ge substrate due to the centrifugal force. With the Ge substrate placed perpendicularly to the centrifugal force, a uniform film of AuNPs could be deposited. The deposited AuNPs film might be more than one layer, and the substrate could be immersed in water to remove the upper layers. A single layer of AuNP could then be obtained. FESEM photographs of AuNPs on Ge obtained by solutions with two different pH values are shown in Figure 2a, b. To estimate the coverage, the color scales of the FESEM pictures were first transformed to gray levels using the commercial graphics tool, Photoshop. Then, we adjusted the contrast of the photograph to get only two values, black and white, where the parts covered by AuNPs was represented by the color white. Afterward, we used Matlab to transform the photograph into a binary bitmap. The black blocks were represented by the digit zero while white ones were represented by the digit one in the binary bitmap. Finally, the respective numbers of ones and zeros were counted and calculated to obtain the ratio of the number of ones to the total amount, which corresponded to the coverage ratio. Figure 2a shows the sample of ‘10.5% AuNPs,’ which was prepared by the AuNP solution with a pH value of 4, and the coverage ratio of AuNPs was estimated to be ~10. 5% on Ge. If the pH value was only slightly decreased, the distribution of AuNPs on Ge became much denser, as shown in Figure 2b, with the ‘30.3% AuNPs’ sample possessing an AuNP coverage ratio of 30.3%. The pH value of the 30.3% case was very close to 4, but it should be slightly smaller than 4, if it is to result in a larger coverage density. Any small variation or error would result in a large variation in coverage. Hence, FESEM imaging is a significant tool for determining the final coverage ratio after AuNP deposition. The AuNPs on 10.5% AuNPs is highly dispersive, and AuNPs start to aggregate on 30.3% AuNPs.

### Photodetector fabrication

Circular Al gates with areas of 5 × 10^-4^ cm^2^ were then deposited on top of Ge substrates to form the photodetectors. Large-area C-shaped Al was also deposited to act as surrounding ohmic contacts at the same time. In order to identify the effect contributed by the AuNPs, the control sample (control) without AuNPs was also prepared, for comparison.

### Photoresponse measurement

**Figure 3:**
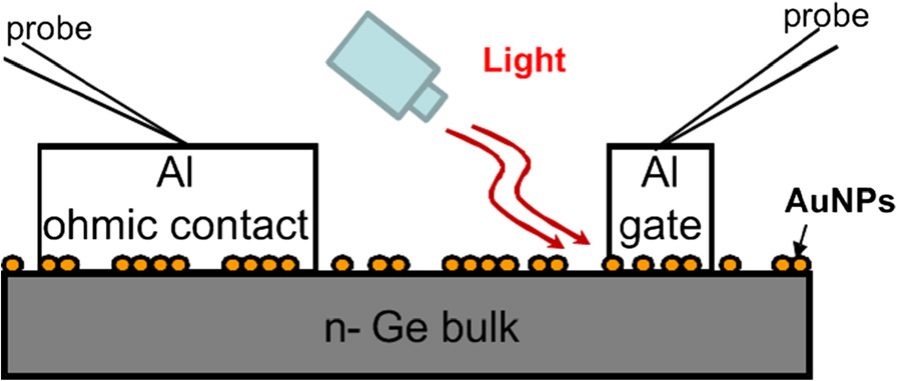
**The structure of the n-type Ge photodetector with AuNPs covering it and the setup used to measure the photoresponse.** The tip of the fiber was tilted at 45 degrees and pointed toward the edge of the circular Al gate with an optical power of 1.5 mW.

The schematic structure of an n-type Ge photodetector with AuNPs and the setup used to measure the photoresponse are shown in Figure 3. The laser diode with an emission wavelength at 1,310 nm and an optical power of 1.5 mW was irradiated at the edge of the circular Al gate to measure the photoresponse. The core size of the fiber output of the laser diode is 9 μm, which is much larger than each nanoparticle (~30 nm), and hence, the measured optical characteristics would be contributed by the abundant AuNPs.

## Results and discussion

To find the maximum response, we continuously moved the tip of the fiber output inward into the Al gate and measured the respective photocurrents. The alternate photocurrents and dark currents were measured until the representative current levels were obtained. It was found that the photocurrents gradually increased to the saturated maximum as the tip of the fiber output was moved inward into the gate and then decreased due to the blockage of light by the electrode. Hereafter, we only show the representative maximum photocurrent and the representative dark current.

**Figure 4:**
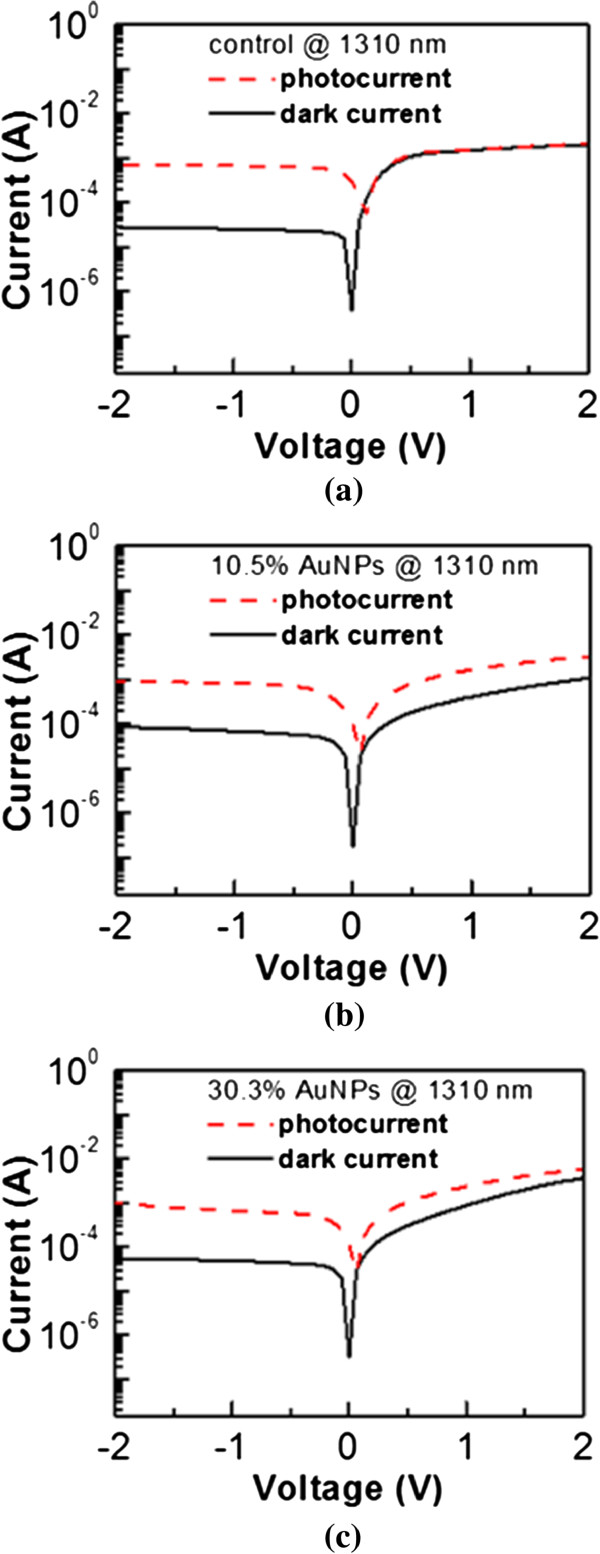
**The dark currents and photocurrents of (a) control, (b) 10.5% AuNPs, and (c) 30.3% AuNPs samples.** The 1,310-nm responsivities at -2 V of the control, 10.5% AuNPs, and 30.3% AuNPs samples are 465, 556, and 623 mA/W, respectively.

**Figure 5:**
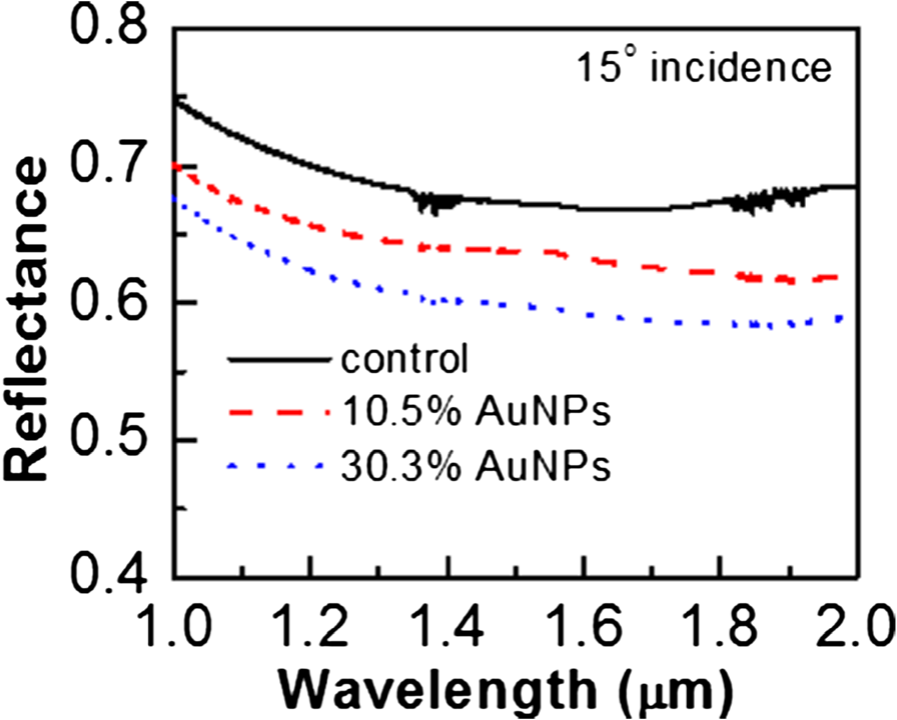
**The reflectance spectra of control, 10.5% AuNPs, and 30.3% AuNPs samples.** The deposition of AuNPs can indeed decrease the reflection of incident light, and a larger coverage ratio results in a smaller reflection.

The current–voltage (I-V) curves of the control, 10.5% AuNPs, and 30.3% AuNPs samples are shown in Figure 4a, b, c, respectively. For photodetectors with rectifying-diode behaviors, photodetectors are always operated at the reverse bias with a lower dark current. Since the semiconductor, Ge in our study, is n-type, a negative bias applied to the gate is corresponding to a reverse bias. The photocurrent and dark current of the control are 7.25 × 10^-4^ and 2.82 × 10^-5^ A at -2 V, respectively (Figure 4a). The responsivity of the control is therefore 465 mA/W since the output optical power of the laser diode is 1.5 mW. When n-Ge is covered by 10.5% AuNPs, the photocurrent and dark current become 9.2 × 10^-4^ and 8.64 × 10^-5^ A at -2 V, respectively (Figure 4b), and the responsivity can achieve 556 mA/W. As compared to the responsivity of 465 mA/W of the control sample, the responsivity of 556 mA/W of 10.5% AuNPs increases 20% (i.e., (556–465)/465 = 20%). When the AuNP coverage ratio increases to 30.3%, the photocurrent is further increased to 9.9 × 10^-4^ A, while the dark current is 5.5 × 10^-5^ A at -2 V (Figure 4c). Therefore, the 30.3% AuNPs sample has a responsivity of 623 mA/W. AuNPs can increase the responsivity of photodetectors, and a denser coverage ratio results in a larger responsivity. As mentioned, the enhancement of responsivities may be contributed by the forward scattering of plasmon resonant nanoparticles. In order to confirm that the forward scattering indeed occurs, the reflectance spectra of the control, 10.5% AuNPs, and 30.3% AuNPs samples are compared and shown in Figure 5. The reflectance spectrum is measured by reflective Fourier transform infrared (FTIR) spectroscopy with an incident angle of 15° relative to normal incidence. The reflectance at 1,310 nm of the control, 10.5% AuNPs, and 30.3% AuNPs samples are 0.68, 0.65, and 0.61, respectively. The deposition of AuNPs can indeed decrease the reflection of incident light, and a larger coverage ratio results in a smaller reflection. More light can be absorbed by the sample with denser AuNPs, which contributes to the enhancement of responsivities.

**Figure 6:**
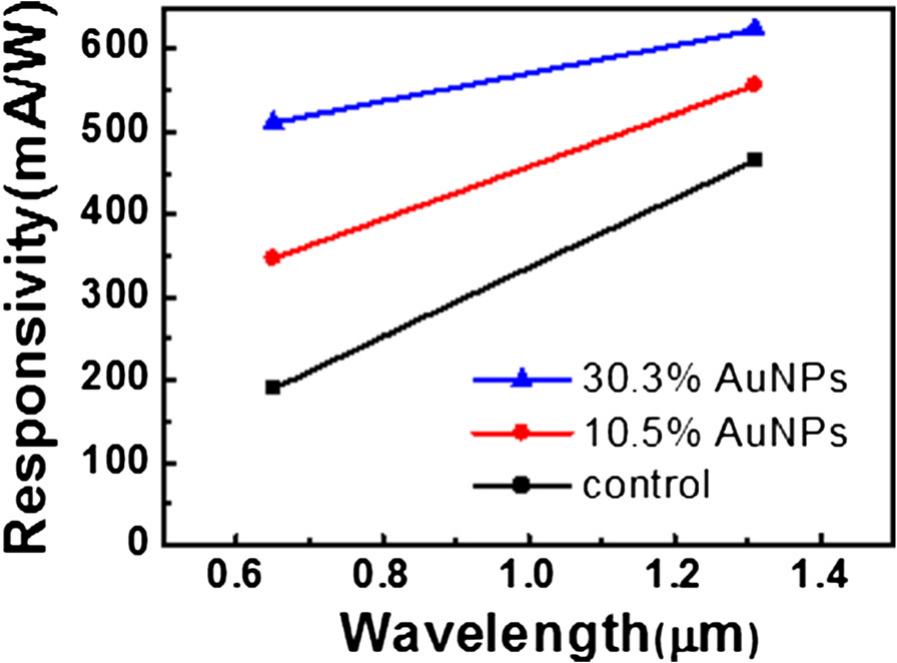
**The responsivity spectra of control, 10.5% AuNPs, and 30.3% AuNPs samples.** The effective input power at 650 nm is estimated to be ~2.1 mW.

Reflectance or photocurrent spectra in ref. [16,17] were measured to show the effects of plasmon resonance. We have measured the reflectance spectra as shown in Figure 5. Regarding the photocurrent spectra, we have also measured the photocurrent at the wavelength of 650 nm and combined with the 1,310-nm results to sketch the approximate responsivity (directly related to the photocurrent) spectra (Figure 6). With denser AuNPs covering, the reflectance can decrease and the responsivity can increase for a wide range of wavelengths. Such experiment results support the photocurrent contribution of AuNPs on Ge.

## Conclusions

The n-Ge photodetectors with and without AuNPs covering them are compared. The deposition of AuNPs can decrease the reflectance of near-infrared via the plasmon resonant nanoparticles. With a coating ratio of 10.5% AuNPs, the reflectance can decrease 4.4%, and the responsivity increases 20% as compared to the control sample. If the coverage ratio of AuNPs increases to 30.3%, the reflectance can decrease 10%, while the responsivity increases 34%. AuNPs is obviously beneficial for Ge near-infrared photodetection.

## Competing interests

The authors declare that they have no competing interests.

## Authors’ contributions

HTH and WFL performed the detector fabrication and the measurement. ICN and SDT assisted with the deposition of AuNPs and provided the discussion of the properties of AuNPs. CHL conceived the experimental plan, modified the process flow, and prepared the manuscript. All authors read and approved the final manuscript.
